# An Introduction to Ventra: A Programmable Abdominal Phantom for Training, Educational, Research, and Development Purposes

**DOI:** 10.3390/s24165431

**Published:** 2024-08-22

**Authors:** Salar Tayebi, Robert Wise, Ashkan Zarghami, Wojciech Dabrowski, Manu L. N. G. Malbrain, Johan Stiens

**Affiliations:** 1 Department of Electronics and Informatics, Vrije Universiteit Brussel (VUB), 1050 Brussels, Belgium; ashkan.zarghami@vub.be (A.Z.); jstiens@etrovub.be (J.S.); 2 Adult Intensive Care, John Radcliffe Hospital, Oxford University Hospitals Trust, Oxford OX3 7LE, UK; rob.wise@ouh.nhs.uk; 3 Discipline of Anaesthesia and Critical Care, School of Clinical Medicine, University of KwaZulu-Natal, Durban 4000, South Africa; 4 Faculty of Medicine and Pharmacy, Vrije Universiteit Brussel (VUB), 1090 Brussels, Belgium; 5 First Department of Anaesthesiology and Intensive Therapy, Medical University of Lublin, 20-090 Lublin, Poland; wojciech.dabrowski@umlub.pl (W.D.); manu.malbrain@telenet.be (M.L.N.G.M.); 6 Medical Data Management, Medaman, 2440 Geel, Belgium; 7 International Fluid Academy, 3360 Lovenjoel, Belgium

**Keywords:** intra-abdominal pressure, gastric pressure, bladder pressure, simulation, human phantom, abdominal compliance, abdominal pressure variation

## Abstract

Background: Intra-abdominal pressure (IAP) is a critical parameter in the care of critically ill patients, as elevated IAP can lead to reduced cardiac output and organ perfusion, potentially resulting in multiple organ dysfunction and failure. The current gold standard for measuring IAP is an indirect technique via the bladder. According to the Abdominal Compartment Society’s Guidelines, new measurement methods/devices for IAP must be validated against the gold standard. Objectives: This study introduces Ventra, an abdominal phantom designed to simulate different IAP levels, abdominal compliance, respiration-related IAP variations, and bladder dynamics. Ventra aims to facilitate the development and validation of new IAP measurement devices while reducing reliance on animal and cadaveric studies. Additionally, it offers potential applications in training and education for biomedical engineering students. This study provides a thorough explanation on the phantom’s design and fabrication, which provides a low-cost solution for advancing IAP measurement research and education. The design concept, technical aspects, and a series of validation experiments determining whether Ventra is a suitable tool for future research are presented in this study. Methods: Ventra’s performance was evaluated through a series of validation tests using a pressure gauge and two intra-gastric (Spiegelberg and CiMON) and two intra-bladder (Accuryn and TraumaGuard) pressure measurement devices. The mean and standard deviation of IAP recordings by each device were investigated. Bland–Altman analysis was used to evaluate bias, precision, limits of agreement, and percentage error for each system. Concordance analysis was performed to assess the ability of Ventra in tracking IAP changes. Results: The phantom demonstrated excellent agreement with reference pressure measurements, showing an average bias of 0.11 ± 0.49 mmHg. A concordance coefficient of 100% was observed for the phantom as well. Ventra accurately simulated different abdominal compliances, with higher IAP values resulting in lower compliance. Abdominal volume changes showed a bias of 0.08 ± 0.07 L/min, and bladder fill volume measurements showed an average difference of 0.90 ± 4.33 mL for volumes ranging from 50 to 500 mL. Conclusion: The validation results were in agreement with the research guidelines of the world abdominal society. Ventra is a reliable tool that will facilitate the development and validation of new IAP measurement devices. It is an effective educational tool for biomedical engineering students as well.

## 1. Introduction

Intra-abdominal pressure (IAP) as the steady-state pressure within the abdominal compartment has been acknowledged as a vital sign in critically ill patients [1,2], gaining a significant amount of attention due to the high prevalence and incidence rates of intra-abdominal hypertension (IAH) in critically ill patients [3,4,5,6,7,8,9]. IAP plays a crucial role in the perfusion pressure of abdominal organs since IAH (defined as sustained IAP equal to or higher than 12 mmHg) increases the back pressure at the venous side and reduces venous return. Subsequently, the low cardiac venous return results in lower cardiac output and organ perfusion, which can lead to multiple organ dysfunction and failure [10,11,12,13]. Following the recognition of IAH as a silent thread to intensive care unit (ICU) patients, various techniques and devices have been introduced to measure IAP, aiming to enhance the current gold standard (via the bladder) and improve patient care [14]. Notable advancements include new catheter-based IAP sensing technologies, such as the Serenno special IAP-sensing device [15,16] and the balloon-in-balloon catheter design of TraumaGuard [17], which enable continuous IAP monitoring. Additionally, the development of ultrasound-based [18], microwave-based [19,20,21], and tensiometry-based [22] IAP-monitoring systems represents progress in creating non-invasive measurement methods to minimize the risk of urinary tract infections and trauma, further advancing the current gold standard.

According to the research guidelines of the Abdominal Compartment Society (WSACS, www.wsacs.org, https://wsacs.mn.co, accessed on 21 July 2024), every novel IAP measurement method/device should be strictly validated against the actual IAP measurement gold standard technique, which is currently an indirect measurement method through the bladder [23,24]. Clinical studies provide the highest level of evidence and insight about a recently introduced measurement technique when it comes to validation studies, but they are often expensive and not always feasible for investigating novel concepts. Preclinical experiments involving cadaveric or animal specimens encounter various challenges. Human cadaveric tissue availability is limited, and ethical concerns surround animal experiments. Moreover, it is not always possible to simulate different case scenarios in animals or cadavers to study the outcome of an IAP measurement device under specific conditions (e.g., respiration maneuvers, significantly high IAP values, variable abdominal compliance, and high body temperature). Developing phantoms to mimic the human body in different situations is a potential solution for the abovementioned issues. In addition to advancing the validation studies for new medical equipment, it will reduce the need for animal studies and avoid potential clinical ethical concerns. Alongside validation studies, human phantoms serve as effective tools for educational purposes as well.

Abdominal phantoms have been previously used for medical imaging research and device development [25,26,27], radiotherapy dosimetry studies [28,29,30,31], hernia repair [32], laparoscopic and robotic surgery training [33,34,35,36], abdominal sonography training [37,38], and abdomen biomechanical studies [39]. Nevertheless, no abdominal phantom has been developed to mimic the abdominal compartment for IAP measurement research and training purposes. In this study, we introduce Ventra, an abdominal phantom that can simulate different IAP and abdominal compliance levels, respiration-related IAP variations, and bladder input and urine output values. It is compatible with intra-bladder pressure (IBP) and intra-gastric pressure (IGP) measurement devices for validation purposes. This innovative abdominal phantom addresses a gap in this domain, since no abdominal phantom has previously been developed for IAP measurement and urine output studies. Training and education are other potential applications of Ventra for biomedical engineering students to develop sensor network systems to measure and analyze biomedical signals. The phantom’s design and fabrication, together with the validation study on its performance, are presented in this paper.

The primary research objective of this study is to introduce and assess the functionality and performance of Ventra for validating new IAP measurement methodologies and devices prior to clinical studies. The goal is to determine, through various experimental and statistical analyses, whether this phantom is suitable for use in future validation studies. The outcomes of this research may offer valuable insights into the advantages and limitations of IAP measurement devices, potentially leading to the development of more efficient, accurate, and reliable technologies for IAP screening in the long term.

## 2. Materials and Methods

### 2.1. Abdominal Compartment

**Figure 1:**
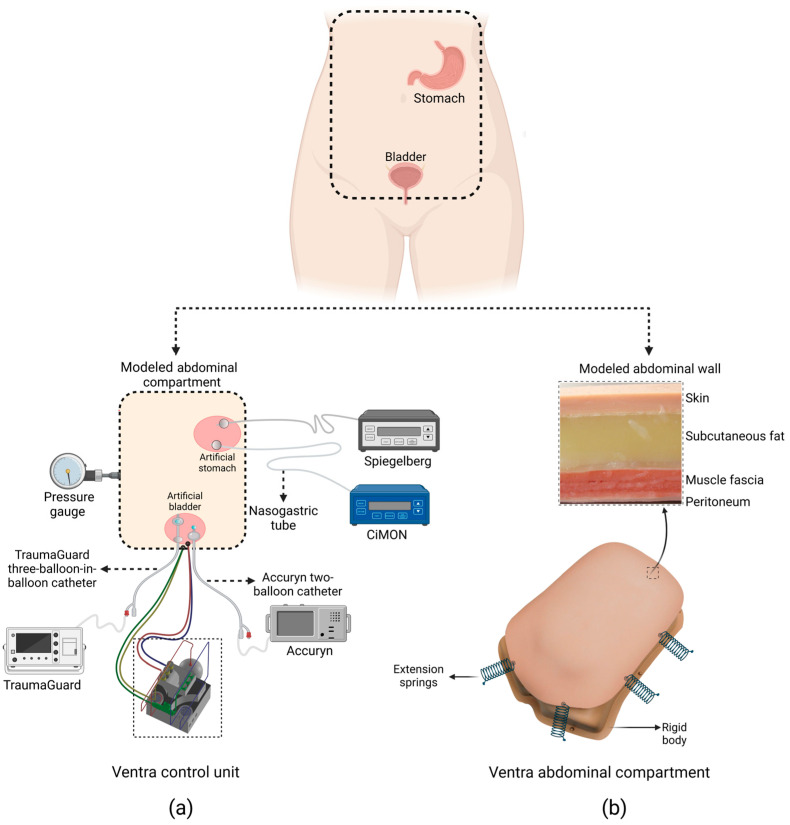
An illustration of the abdominal compartment and abdominal wall of the phantom. (**a**) Two sub-compartments were placed inside the modeled abdominal compartment to represent the bladder and stomach. Two intra-bladder and two intra-gastric pressure measurement instruments were connected to the abdominal compartment as well. (**b**) An artificial abdominal wall including skin, fat, muscle, and peritoneum was placed on top of the abdominal compartment. By connecting the abdominal wall to the ground surface by extension springs, the abdominal compliance can be adjusted further.

The abdominal compartment was modeled as an inflatable balloon with a total volume of 4000 mL and made of butyl rubber (TER Chemicals GmbH & Co., Hamburg, Germany). The bladder and stomach were modeled and fabricated as two sub-compartments using 500 mL and 1000 mL styrene ethylene butylene balloons (Dahlhausen Inc., Bochum, Germany) and placed inside the abdominal compartment. Subsequently, a two-balloon Foley catheter (Accuryn, Potrero Medical, Hayward, CA, USA) and a three-balloon-in-balloon Foley catheter (TraumaGuard, Sentinel Medical Technologies, Jacksonville, FL, USA) were connected to the bladder compartment for IBP measurement. Two nasogastric tubes were connected to the stomach to make the phantom compatible with IGP measurements as well. The nasogastric tubes were connected to CiMON (former Pulsion Medical Systems, now integrated within Getinge, Sölna, Sweden) and Spiegelberg (Spiegelberg, Hamburg, Germany) for IGP measurement. In order to obtain the reference IAP, a pressure gauge (HEINE Optotechnik GmbH & Co., Gilching, Germany) was also connected to the abdominal compartment (see Figure 1a). An artificial abdominal wall (The Chamberlain Group, Great Barrington, MA, USA) was placed on top of the designed abdominal compartment, which represents the abdominal compartment in supine position to be in agreement with the current guidelines on IBP measurement [24]. This multilayer abdominal wall features artificial skin (4 mm thick), subcutaneous fat (13 mm thick), muscle with fascia (10 mm thick), and peritoneum (less than 1 mm thick). These thicknesses are generally consistent with real-world data on abdominal wall layer thicknesses [40,41], which report average values of 2.5 mm for skin, 15 mm for fat, 10 mm for muscle, and 0.5 mm for peritoneum. The abdominal wall was connected to a rigid body using extension springs to make it secure. The abdominal compartment was then placed below the abdominal wall (see Figure 1b). By changing the stiffness of the extension springs, the ease of abdominal expansion can be changed, which changes abdominal compliance defined as changes in the abdominal volume divided by the changes in end-expiration IAP (ΔIAV/ΔIAP_ee_). For this study, the stiffness of the springs could be fine-tuned to simulate an abdominal compliance value in agreement with previously reported values, when there is no muscular contraction [42]. The developed abdominal compartment was then connected to the control unit, which controls the pressure, bladder, and respiration functions of Ventra.

### 2.2. Control Unit

#### 2.2.1. Electronic Circuit Design and Components

**Figure 2:**
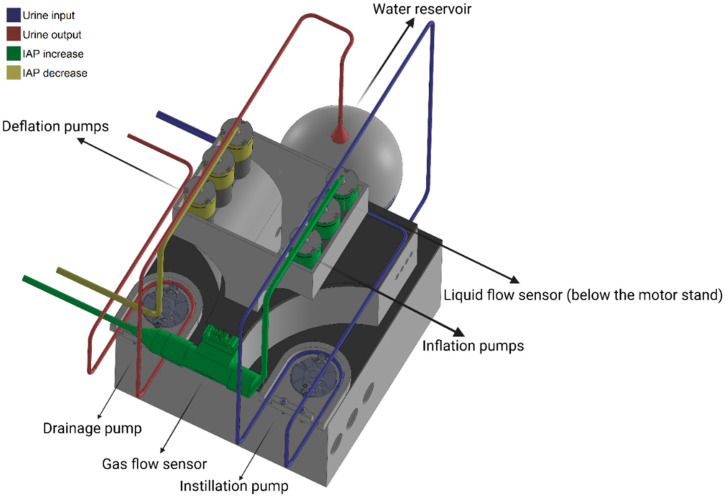
The computer-aided design (CAD) of the enclosure of the phantom.

The electronic circuit was designed using Altium PCB design software (Altium Inc. San Diego, CA, USA) and printed by JLCPCB (JLCPCB Co., Shenzhen, China). The electronic circuit and the printed circuit board (PCB) are presented in ESM Figure S1. Vacuum pumps (Adafruit Industries, New York, NY, USA) were used to inflate/deflate the abdominal compartment and increase/decrease the IAP by gas instillation. This mechanism of IAP elevation and reduction represents the original pathology behind IAP elevation. The gas employed to inflate or deflate the abdominal compartment was regular air. The output of the pumps was connected to a thermal gas flow sensor (Honeywell Inc., Charlotte, NC, USA) to monitor the gas volume instilled inside the abdominal compartment. This sensor is crucial to record intra-abdominal volume changes (ΔIAV). A pressure transducer (Panasonic Industry Europe GmbH., Hamburg, Germany) was also used to measure IAP for the settings of Ventra. The pressure transducer was zeroed with respect to the IBP catheter’s tip height level to prevent any bias due to the water height difference between the IBP catheter’s tip and the pressure transducer. Since the catheter sensor balloons are air-charged, the height of the catheter’s tip and pressure transducer within the abdominal compartment do not make any difference to IAP readings. Therefore, all the IAP recordings are assumed to represent the IAP at the mid-axillary line at the iliac crest level (in compliance with the current gold standard [24]). Two peristaltic pumps were also used to instill and remove fluid to and from the modeled bladder. The fluid utilized to replicate the bladder fill volume and urine output was distilled water. The output of the fluid pumps was also connected to a turbine fluid flow sensor (Honeywell Inc., Charlotte, NC, USA) to monitor the fluid volume being inserted into the bladder, which resembles the urine input volume. Using the information from pressure and flow (gas and liquid) sensors, and a microcontroller (Arduino Inc., Somerville, MA, USA), the phantom was programmed and controlled. Lastly, a computer-aided design (CAD) of the enclosure for the components was designed in SolidWorks (SolidWorks Corp., Waltham, MA, USA) and 3D printed by Prusa printers (Prusa research, Prague, Czech Republic). The final assembly is shown in Figure 2.

#### 2.2.2. Graphical User Interface

**Figure 3:**
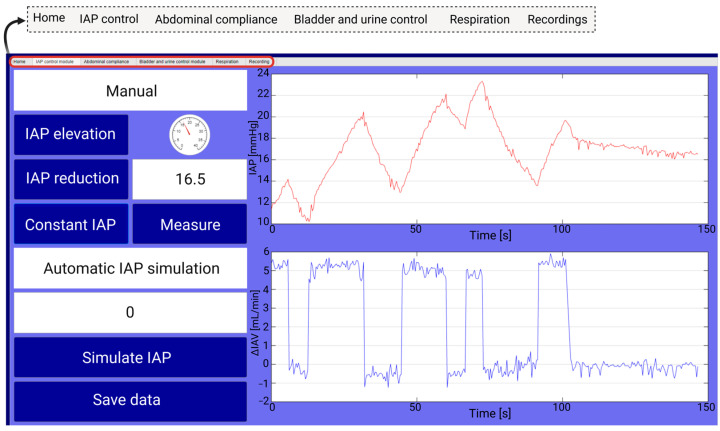
The IAP control module of the GUI. The IAP can be increased, decreased, and kept constant through the IAP control table. An automatic IAP simulation is also designed to deliver the requested IAP value.

As the next step, a graphical user interface (GUI) was made in MATLAB (MATLAB, Natick, MA, USA). As presented in Figure 3, the GUI consists of six general tabs. The IAP control tab is responsible for IAP elevation and reduction. Two different modules were designed and programmed for the IAP control. The first module is a manual panel to increase, decrease, and keep the pressure steady inside the abdominal phantom. The second panel, however, allows us to automatically obtain a certain IAP by inserting its value in the GUI. The continuous IAP and ΔIAV will be monitored and stored here as well.

The abdominal compliance tab is used to run a compliance maneuver and calculate the abdominal compliance [42]. This panel is programmed to start inflating the abdominal compartment and measuring the ΔIAV and IAP changes over time intervals of 4 s. Subsequently, the abdominal compliance can be calculated as ΔIAV/ΔIAP_ee_. The IAP, ΔIAV, and compliance data are plotted and stored continuously as well (see ESM Figure S2).

The urine input/output can be controlled in the bladder and urine control tab. The bladder fill volume can be recorded and stored in this panel. Simultaneously, IAP will be measured and plotted to monitor the IAP when the bladder fill volume changes (see ESM Figure S3).

The respiration tab is responsible for simulating the respiration impact on the abdominal compartment. In this panel, respiration can be controlled by inserting the intended respiration rate or by inserting the desired abdominal pressure variation (APV), defined as the difference between the end-inspiration IAP (IAP_ei_) and the end-expiration IAP (IAP_ee_) value, or ∆IAP, divided by the mean IAP and is expressed as a percentage [42]. Mean IAP, IAP_ei_, IAP_ee_, and APV will be recorded and stored as well (see ESM Figure S4).

Lastly, all the recorded data (numerical values) are stored in separate tables in the recording tab for further data processing and curation and can be exported as csv or txt files (see ESM Figure S5).

### 2.3. Validation Tests

In order to evaluate the functionality and performance of Ventra, several validation tests were designed and run. In the first stage, the robustness of the phantom in simulating different IAP values was examined. The pressure readings by the Ventra, in addition to the IAP readings by two IGP and two IBP measurement devices, were recorded and validated against the pressure readings by the pressure gauge (the reference pressure). Seventy-two measurements were performed at IAP values between 0 and 25 mmHg (with a gradual increase in steps of 5 mmHg). This validation test assessed the accuracy and reliability of the phantom in producing various IAP values. The ability of the phantom to simulate different abdominal compliance values was evaluated by changing the overall stiffness of the springs connecting the abdominal wall to the rigid body. Five measurements were performed at each compliance value to observe the abdominal phantom’s response for the IAP range of 5 to 25 mmHg. The goal of this test was to examine the compliance curves that Ventra can produce. The ΔIAV was assessed and validated against an analog gas flow meter. Five measurements were performed at flow rates of 1, 2, 3, and 4 L/min to check the accuracy and reliability of the IAV measurements, which indirectly determine the abdominal compliance. To evaluate and validate the bladder fill volume, five measurements were performed at 50 to 500 mL (steps of 50 mL). At the beginning of each experiment, the water volume inside the water reservoir was measured manually. Afterwards, the recorded volumes with the flow sensor were compared and validated with the water reservoir volumes. This test was vital to assess the accuracy of fluid volume monitoring inside the bladder, which can potentially have a significant impact on IBP measurement devices. Lastly, ten respiration cycles with APV values of 10%, 20%, and 30% were performed with the phantom to observe its respiration function in making dynamic IAP changes and to evaluate the phantom’s accuracy in simulating different respiration rates and APVs.

The validation experiments were based on several assumptions. The chosen IAP, compliance, IAV, and respiration-related values were intended to represent real-world data. However, the rate of IAP changes was significantly higher than what is typically observed, challenging the equipment’s ability to detect an IAP elevation or reduction quickly in the phantom. The temperature of the phantom was kept constant at 25 °C to avoid any data variation due to temperature fluctuations. Nonetheless, elevated temperatures, simulating fever in patients, can be achieved by introducing higher-temperature water or gas into the phantom. This feature allows for the evaluation of the robustness of novel IAP measurement technologies in patients with fever.

### 2.4. Data Processing and Assessment

Results are presented as mean and standard deviation. A paired-samples t-test was performed between the IAP_Gauge_ and IAP_Ventra_ and other IBP and IGP devices to determine the significance of the difference between them. A *p*-value smaller than 0.05 was considered significantly different. Bland–Altman analysis was done to calculate the bias in IAP measurements, defined and calculated as the mean difference between the IAP_Ventra_ and the IAP_Gauge_. Calculating bias will clarify the systematic difference between the measurements. Subsequently, the precision and limits of agreement between IAP_Ventra_ and IAP_Gauge_ were defined as the standard deviation of the bias and the bias ± 1.96 times the precision, respectively, according to Bland and Altman [43]. The percentage error was then calculated as the precision multiplied by 2 and divided by the mean IAP value of each piece of technology to examine the relative accuracy and agreement between two measurement methods. The capability of Ventra to monitor changes in IAP over time was assessed using concordance analysis. Specifically, the ΔIAP_Ventra_ was compared to the ΔIAP_Gauge_ within the same time period. The concordance coefficient was defined as the percentage of pairs that exhibited the same direction of change. This calculation excluded pairs where both ΔIAP_Ventra_ and ΔIAP_Gauge_ were ≤2.5 mmHg (or less than 15% change) as well as pairs where either ΔIAP_Ventra_ or ΔIAP_Gauge_ was zero [44]. This metric determines the agreement between two measurement methods in terms of their ability to consistently reflect changes, rather than their absolute values. Except for the concordance analysis, the abovementioned statistical evaluations were adapted and performed on abdominal and bladder fill volume data, too. Subsequently, a thorough assessment of the recorded data was conducted to ensure that the data maintained their integrity throughout the study. The dataset was found to be complete, accurate, and consistent. Therefore, no additional data cleaning procedures were required.

## 3. Results

### 3.1. Phantom Setup

**Figure 4:**
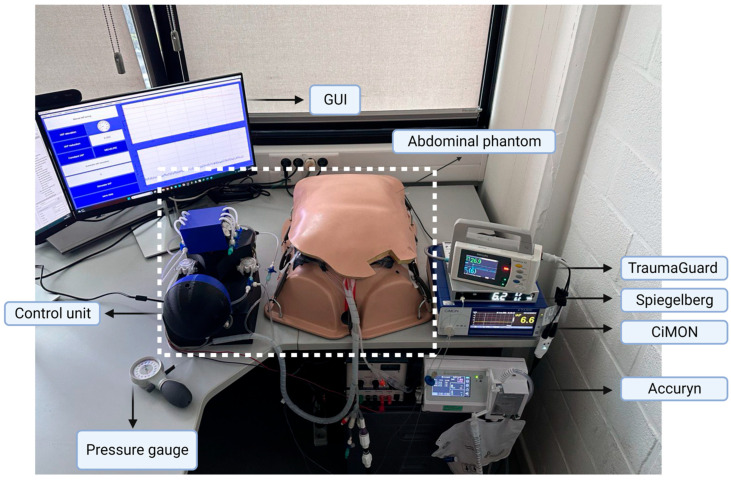
The phantom setup. The phantom, its control unit, and its GUI in addition to the intra-bladder and intra-gastric measurement devices measuring an IAP of 7 mmHg.

The Ventra is presented in Figure 4. As can be seen, the abdominal phantom is connected to the control unit and intra-bladder and intra-gastric pressure measurement devices. The GUI can be seen as well.

### 3.2. Intra-Abdominal Pressure Simulation

**Figure 5:**
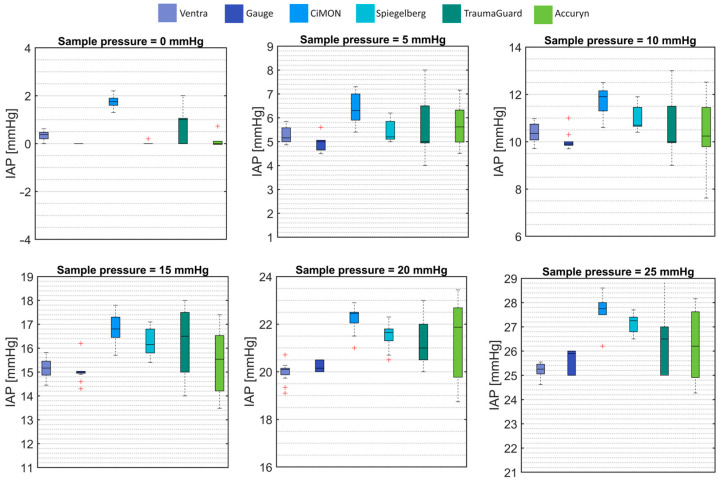
The mean and standard deviation of the IAP measurements at 0, 5, 10, 15, 20, and 25 mmHg. The error bars are the 95% confidence interval, the bottom and top of the box are the 25th and 75th percentiles, respectively, the line inside the box is the 50th percentile (median), and any outliers are shown in red.

As the first technical validation analysis, the phantom’s ability to simulate different IAP values was examined. The mean and standard deviation of IAP recordings by each system are presented in Figure 5 and Table 1.

In general, the intra-gastric devices showed a slight overestimation of the IAP inside the phantom. However, the Ventra and intra-bladder equipment showed results in agreement with the IAP gauge.

**Figure 6:**
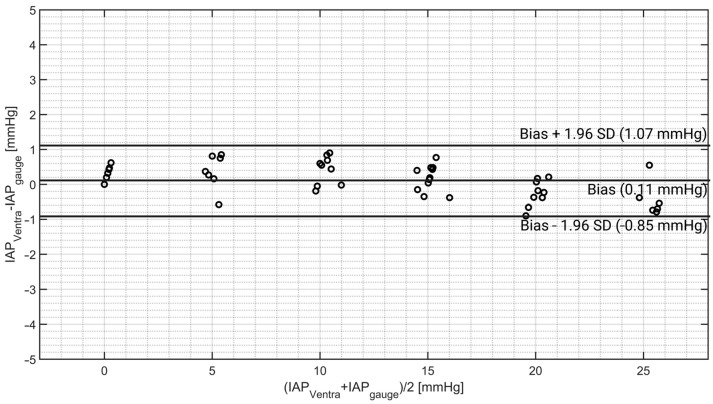
Bland–Altman results of the simulated IAP. Ventra showed a slight bias of +0.11 mmHg when compared with the pressure gauge.

The Bland–Altman results are presented in Figure 6.

It can be observed that at IAP values smaller than 15 mmHg, Ventra shows a slight overestimation of the pressure; however, at IAP values higher than 15 mmHg, the overestimation becomes smaller and in some cases underestimation. The numeric value of the Bland–Altman analysis of each system is presented in Table 2.

Reviewing the Bland–Altman results, TraumaGuard and Accuryn showed higher accuracy than CiMON and Spiegelberg. However, the IGP measurement devices showed better precision compared with the IBP measurement devices.

**Figure 7:**
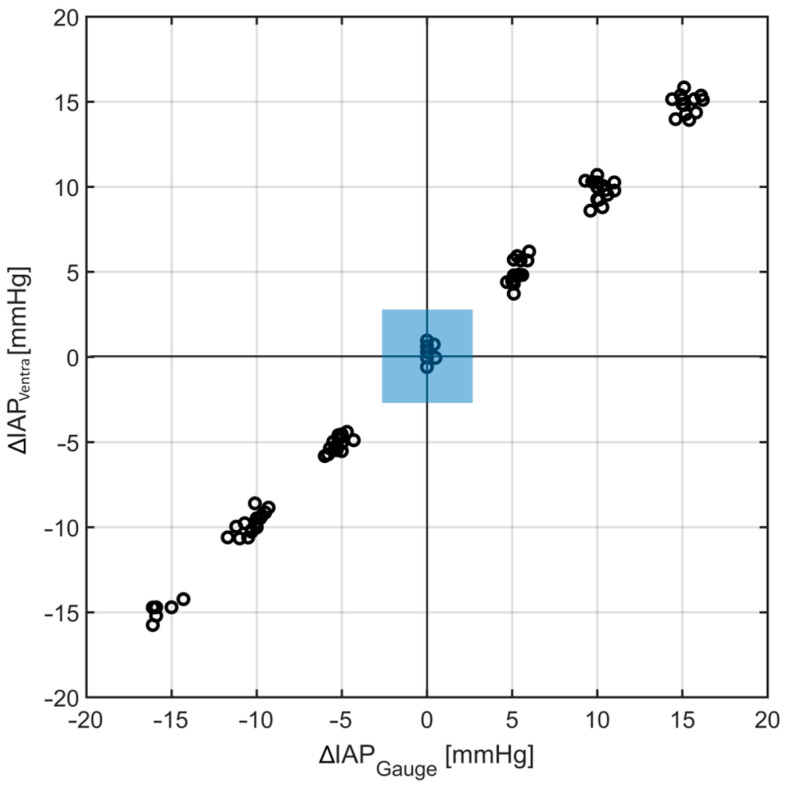
The concordance analysis result. A concordance coefficient of 100% was observed for Ventra, which shows the capability of this abdominal phantom in tracking IAP changes (the blue region indicated the excluded data).

The concordance analysis resulted in a concordance coefficient of 100% (see Figure 7).

### 3.3. Abdominal Compliance

**Figure 8:**
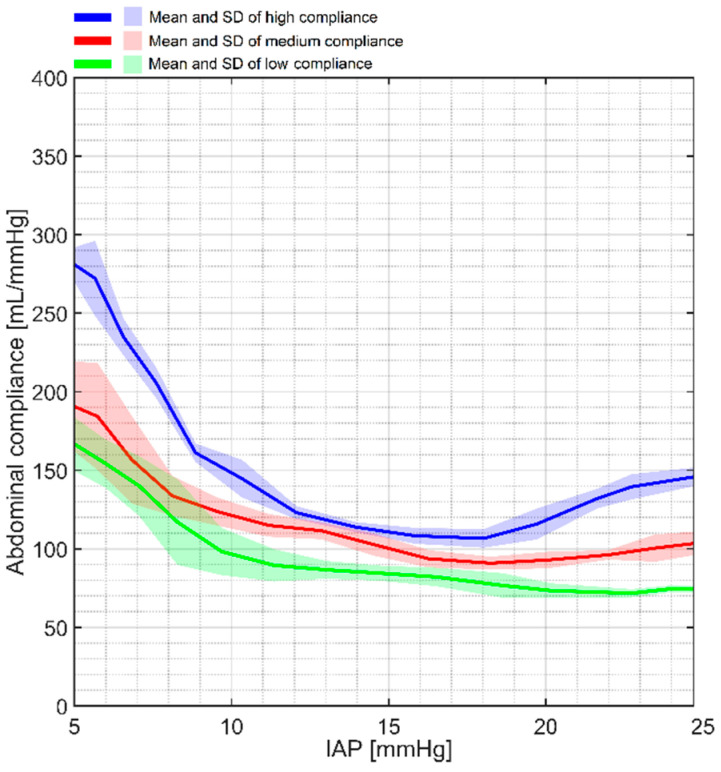
Compliance maneuver of the phantom at high-, medium-, and low-compliance conditions. A relatively high compliance can be observed at IAP values lower than 10 mmHg. However, at IAPs higher than 10 mmHg, the impact of the springs becomes more dominant, which results in reduced compliance.

The compliance results of the phantom at three different conditions are shown in Figure 8.

As presented, the compliance response of the phantom was in agreement with the actual abdominal behavior, where compliance was initially higher and decreased as the IAP increased. The numerical values of compliance at 5, 10, 15, 20, and 25 mmHg are presented in Table 3.

### 3.4. Abdominal Volume Monitoring

**Figure 9:**
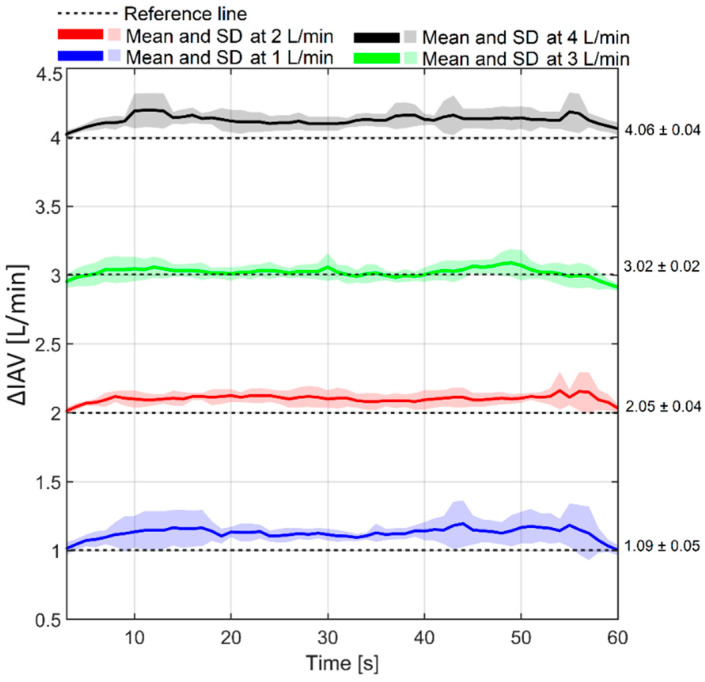
Mean and standard deviation of ΔIAV recorded by the gas flow sensor. The measurements at four different flow rates are compared with the reference measurements by an analog flow meter.

The accuracy of the gas flow sensor was then evaluated and validated. The accuracy of this parameter is also important since it indirectly determines abdominal compliance. The gas flow at four different flow rates is depicted in Figure 9.

**Figure 10:**
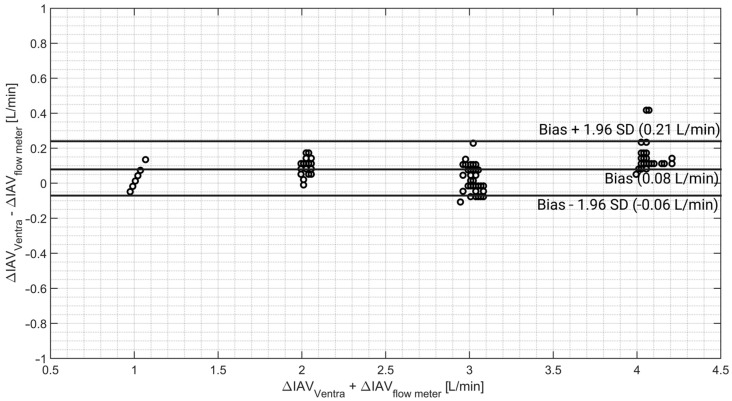
Bland–Altman results of ΔIAV. A relatively small bias was noticed between Ventra and the analog flow meter.

On average, a bias of 0.08 ± 0.07 L/min was observed between the recorded ΔIAV by the gas flow sensor and the reference flow meter. The Bland–Altman results for ΔIAV are shown in Figure 10.

A precision of 0.07 L/min with a ULA and LLA of 0.21 and −0.06 L/min, respectively, was obtained for the ΔIAV measurements. The Bland–Altman analysis also showed a percentage error of 2.7%.

### 3.5. Bladder Fill Volume Monitoring

**Figure 11:**
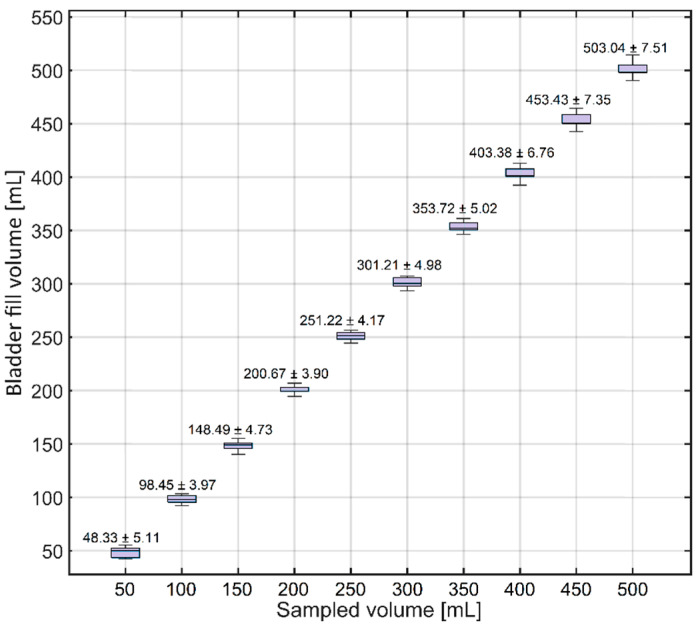
Bladder fluid volume measurement results. The results show an accurate bladder fluid volume measurement.

The monitoring of fluid flow into the bladder was then evaluated. Figure 11 illustrates the mean and standard deviation of the bladder fill volume at intervals of 50 mL.

**Figure 12:**
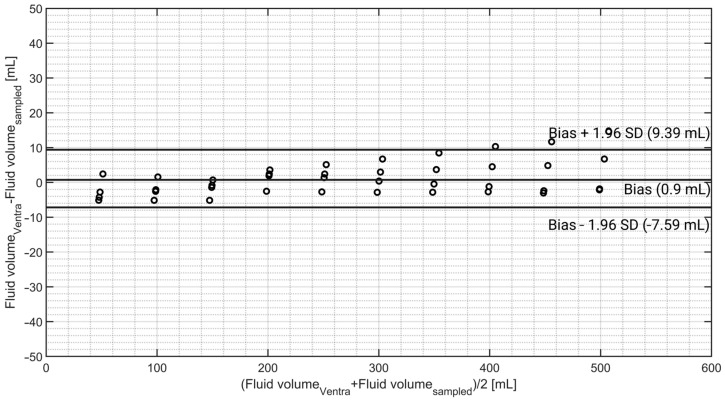
Bland–Altman analysis of the bladder fill volume. As already noticed, an underestimation happens for volumes smaller than 200 mL. However, at higher volumes the underestimation becomes a slight overestimation.

The results show a slight underestimation of the bladder fill volume for fluid volumes smaller than 200 mL. However, at volumes higher than 200 mL, a slight overestimation can be observed in the results. The Bland–Altman results of the bladder fill volume are presented in Figure 12.

Bland–Altman analysis of the bladder fill volume showed bias, precision, limits of agreement, and percentage error values of 0.90 mL, 4.33 mL, −7.59 mL, 9.39 mL, and 3.14%, respectively.

**Figure 13:**
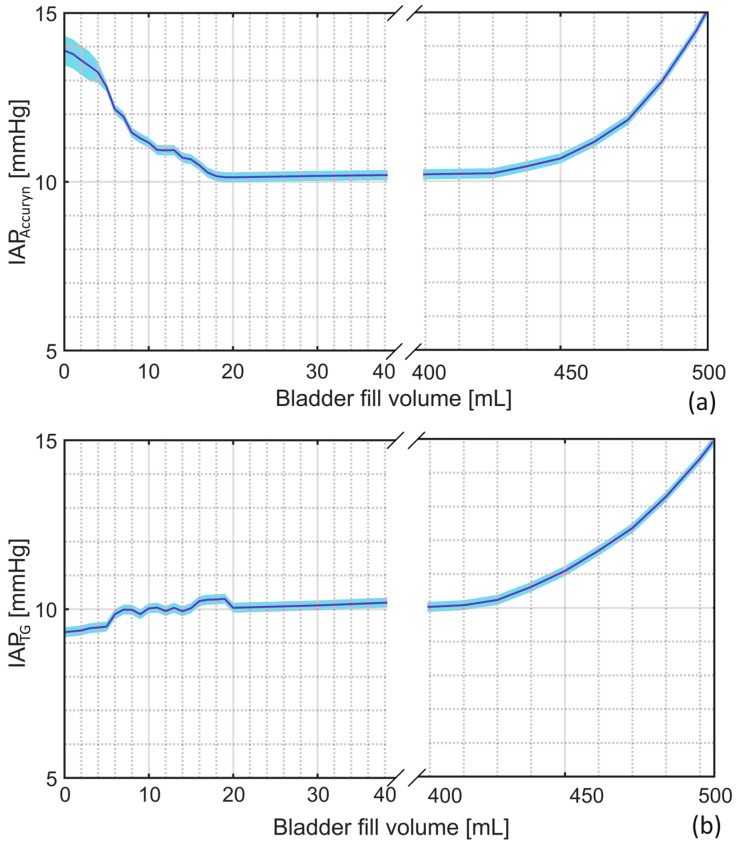
The IAP measurements by the (**a**) Accuryn and (**b**) TraumaGuard catheters in Ventra at different bladder fill volumes.

In order to show the importance of the bladder fill volume in the accuracy of the IBP measurements, the IAP recordings by the TraumaGuard and Accuryn catheters at varying bladder fill volumes are depicted in Figure 13.

As observed, the measurement accuracy when the bladder fill volume is less than 20 mL is smaller compared with the scenarios where the bladder has more than 20 mL of fluid.

### 3.6. Respiration Simulation

**Figure 14:**
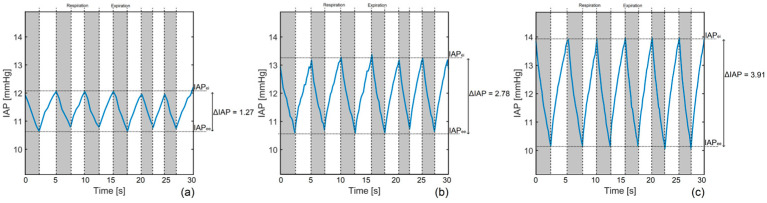
Dynamic IAP changes due to respiration with I:E set at 1:1. IAP changes due to respiration with an abdominal pressure variation of (**a**) 10%, (**b**) 20%, and (**c**) 30%. The end-inspiration (IAP_ei_), end-expiration IAP (IAP_ee_), and ΔIAP are illustrated as well.

As the last validation analysis, the dynamic IAP fluctuations due to respiration were simulated by the phantom. Figure 14 presents the IAP oscillations at three different abdominal pressure variations.

More numerical results of the respiration-related IAP simulations by Ventra are presented in Table 4.

## 4. Discussion

This study aimed to develop an abdominal phantom suitable for validation studies, emphasizing its potential as an educational tool. Human phantoms are valuable in active learning methodologies, which engage learners more actively in the learning process. These tools are crucial for facilitating learning beyond traditional classroom environments, making them particularly beneficial for adult learners in professional settings [42]. In research and education, human phantoms enable the design of various case scenarios and the evaluation of medical equipment performance for diagnosis and treatment purposes [43].

### 4.1. IAP Simulation

The validation results show an excellent agreement between the simulated IAP in the phantom with the IAP values recorded by a pressure gauge (reference pressure), CiMON, Spiegelberg, TraumaGuard, and Accuryn as IGP and IBP measurement methodologies. On average, a bias of 0.11 ± 0.49 mmHg was observed between the phantom and gauge IAP readings, confirming the accuracy of the phantom in simulating different IAP values. A lower limit of agreement, upper limit of agreement, and percentage error of −0.85, +1.07 mmHg, and 7%, respectively, was also observed for Ventra. Regarding the IBP and IGP measurements, however, a bias and precision of −1.70 ± 0.53, −0.84 ± 0.85, −0.77 ± 0.47, and −0.50 ± 0.61 mmHg was observed between Ventra and CiMON, Spiegelberg, TraumaGuard, and Accuryn, respectively, which is coherent with previously reported values [45]. In general, IGP measurement devices provide less accuracy in IAP monitoring compared with IBP systems. Taking the validation results and the research guidelines of the Abdominal Compartment Society into account [23], Ventra can potentially be used to evaluate the robustness of IAP measurement equipment in vitro. In this abdominal phantom, regular air was used to inflate and deflate the abdominal compartment to change the IAP, although CO_2_ is typically used for inflating the abdomen during laparoscopic surgeries since it is colorless, inexpensive, nonflammable, and has higher blood solubility than air [46]. Nevertheless, since there is no biochemical interaction in Ventra, using regular air instead of CO_2_ does not influence the results.

### 4.2. Abdominal Compliance

The abdominal compliance maneuver showed that different compliance values can be generated via the phantom, highlighting one of Ventra’s unique features that sets it apart from existing commercial phantoms. In this study, three different compliances were simulated over an IAP range of 5 to 25 mmHg. There are two main parts that determine the total compliance of the phantom: the elasticity of the abdominal compartment and the stiffness of the extension springs. In the initial part of the compliance plot (for IAPs less than 7 mmHg), the impact of the abdominal compartment’s elasticity is more dominant. However, at higher IAP values (more than 12 mmHg) the impact of the extension springs will be more impactful and influences the total compliance of the phantom. In general, higher IAP values will generate a higher degree of extension in the springs, which generates higher extension forces (proportional to the stiffness coefficient of the springs). Therefore, abdominal compliance will decrease when the IAP goes up. This response is completely representative of the real human body, as discussed in previous studies on abdominal compliance [47], and ΔIAP_ee_ tends to increase for a fixed ΔIAV when the IAP increases. Thus, lower compliance will be experienced at higher IAP values. Reviewing the compliance results revealed a slight compliance increase at very high IAP values (25 mmHg). This can be justified by taking the elastic limit of the springs into account. Extension springs generate tension when elongated within their elastic limits and where the force magnitude is proportional to the elongation. However, when a spring is elongated beyond its elastic limits, it generates less tension compared with the tension generated in the elastic limits. This could be the main reason why compliance goes up slightly after a certain IAP value.

### 4.3. Intra-Abdominal Volume Changes

The phantom’s accuracy in monitoring gas flow rates of 1, 2, 3, and 4 L/min was validated against an analog flow meter. Validation results show a bias of 0.08 ± 0.07 L/min between the reference and recorded values by the sensor. The flow rate accuracy is of great importance as it indirectly determines abdominal compliance. Moreover, the respiration waveform and APV are also a function of the air volume that is inflated into the phantom. Therefore, it is necessary to have an accurate and steady gas flow for simulating IAP elevation/respiration.

### 4.4. Bladder Fill Volume

The capability to monitor and adjust the bladder fill volume is another unique feature of Ventra, allowing it to evaluate the performance of IBP methods at varying bladder fill levels. The bladder fill volume measurement showed excellent results as well. On average, a difference of only 0.90 ± 4.33 mL was observed for fluid volumes in the range of 50 to 500 mL. The bladder fill volume is important in testing the IAP measurement device’s robustness at different bladder fill volumes. The IAP measurement by the TraumaGuard and Accuryn catheters indicates that, when the bladder contains less than 15 mL of fluid, the measurement accuracy is lower compared with when the bladder has at least 15 mL of fluid. In addition to this research, previous studies reveal that different balloon-based pressure catheters might read erroneous IAP values when the bladder fill volume is smaller than 11 mL [41]. This is in complete agreement with the IBP guidelines, which emphasize that an IBP measurement must be performed when 20 mL of saline is instilled into the bladder [24].

### 4.5. Respiration-Related IAP

Simulating respiration-related IAP values, particularly abdominal pressure variation (APV), is another feature of Ventra. In this study, the I:E ratio was set at 1:1; however, it is better to have a ratio of 1:2 to have a more realistic simulation. The I:E ratio can be further adjusted by changing the inspiration and expiration timings in the phantom settings. In addition, the duty cycle of the motors should be adjusted to have proper IAP fluctuations during respiration. The respiration maneuver is another important feature of the phantom as it can determine the robustness of IAP measurement technologies in tracking dynamic IAP fluctuations and provide insights into the response time of the medical devices and their ability to perform continuous IAP monitoring.

### 4.6. Limitations

The present study has several limitations. First, the maximum simulated IAP was 25 mmHg, which is high enough to validate medical devices; however, providing the possibility of generating higher IAP values could be an added value. Second, the IAP fluctuations were induced by gas instillation into the abdominal compartment. While this method of elevating and reducing IAP is representative of laparoscopic surgeries, where IAP is controlled by insufflators, it does not reflect most pathological cases where IAP elevation is typically due to fluid accumulation, which was not simulated in this study [48]. Third, while it was possible to change the compliance between high, medium, and low values, the dynamic range of the compliance could be wider in the future. In this study, the maximum compliance generated was approximately 280 mL/mmHg. Although the human abdominal compliance is within this range, some special scenarios can result in even higher compliance values. Fourth, concerning the bladder fill volume, we used distilled water to simulate urine input and output, which has a different viscosity, surface tension, chemical compositions, and density. These differences in the medium that IBP catheters work with might induce some minor differences. Fifth, regarding the respiration impact on IAP variations, the inflation and deflation motors should be more powerful to simultaneously generate high APVs with high respiration rates. It is possible to simulate any respiration rate with the phantom, and it is also possible to simulate any APV value. However, simulating a specific APV together with a specific respiration rate might be challenging since the actual motors might not be able to generate fast IAP fluctuations. Sixth, simulating the heartbeat is another feature that can be added in future developments of the Ventra. Finally, the absence of a feature to elevate the temperature of the fluid or gas to simulate a patient with fever is another limitation of the current version of Ventra.

### 4.7. Future Developments

In future generations of Ventra, several improvements can be made. First, the capability to instill fluid into the main compartment to alter the IAP through fluid accumulation would more accurately reflect pathological cases. Another technical improvement involves enhancing the control unit programs to manage the duty cycle of the motors, thereby controlling the rate of IAP changes. To improve Ventra’s compliance, adding tunable extension springs could introduce further novelty and control over compliance adjustments. Using urine-mimicking fluids to simulate different bladder fill volumes is another aspect that can be improved for future studies. For instance, by using multi-purpose artificial urine [49], we can enhance Ventra to more closely mimic the properties of real urine. Utilizing stronger vacuum pumps would enhance the simulation of respiration-related IAP, enabling Ventra to simulate any APV with any respiration rate. Concerning the heartrate simulation, mimicking the slight pressure oscillations transmitted to the abdominal compartment from the heartbeat could be achieved by incorporating another vacuum pump to inject and withdraw air from the main compartment at a frequency representative of the heart rate. Lastly, incorporating a small heater to allow Ventra to raise the abdominal compartment’s temperature by increasing the temperatures of the fluid and gas introduced into the phantom is a potential development to consider for future generations.

## 5. Conclusions

The validation outcomes were consistent with the research guidelines established by the world abdominal society. Ventra has proven to be a dependable tool that will aid in the creation and validation of new IAP measurement devices. Additionally, it serves as an effective educational resource for biomedical engineering students.

## Figures and Tables

**Table 1:** Mean and standard deviation of the recorded IAP values via Ventra, the pressure gauge, CiMON, Spiegelberg, TraumaGuard, and Accuryn.

	Phantom		Intra-Gastric		Intra-Bladder	
Sampled IAP (mmHg)	Ventra (mmHg)	Gauge (mmHg)	CiMON (mmHg)	Spiegelberg (mmHg)	TraumaGuard (mmHg)	Accuryn (mmHg)
0 (*n* = 12)	0.34 ± 0.21	0.00 ± 0.00	1.75 ± 0.29	0.03 ± 0.08	0.83 ± 0.72	0.14 ± 0.23
5 (*n* = 12)	5.28 ± 0.35	4.89 ± 0.29	6.41 ± 0.67	5.45 ± 0.43	5.67 ± 1.15	5.68 ± 0.84
10 (*n* = 12)	10.39 ± 0.43	10.01 ± 0.35	11.73 ± 0.56	11.00 ± 0.49	10.58 ± 1.16	10.46 ± 1.38
15 (*n* = 12)	15.18 ± 0.43	15.00 ± 0.44	16.82 ± 0.64	16.24 ± 0.62	16.25 ± 1.49	15.44 ± 1.28
20 (*n* = 12)	19.98 ± 0.42	20.21 ± 0.47	22.25 ± 0.53	21.54 ± 0.55	21.16 ± 0.94	21.40 ± 1.66
25 (*n* = 12)	25.20 ± 0.32	25.63 ± 0.47	27.63 ± 0.76	27.15 ± 0.41	26.51 ± 1.46	26.23 ± 1.43
**Average IAP**						
12.5 (*n* = 72)	12.73 ± 9.25 *p* = 0.94	12.62 ± 9.58 -	14.43 ± 9.73 *p* = 0.26	13.57 ± 10.12 *p* = 0.56	13.50 ± 8.89 *p* = 0.57	13.25 ± 8.99 *p* = 0.69

**Table 2:** The numeric results of the Bland–Altman analysis of IAP.

Device	Mean IAP (mmHg)	Bias (mmHg)	Precision (mmHg)	ULA (mmHg)	LLA (mmHg)	PE (%)
Ventra	13.33	0.11	0.49	1.07	−0.85	7
CiMON	15.03	1.81	0.57	2.93	+0.69	8
Spiegelberg	14.23	1.01	0.63	2.24	−0.22	9
TraumaGuard	14.16	0.94	1.16	3.21	−1.33	16
Accuryn	13.85	0.62	1.52	3.60	−2.36	21

ULA, upper limit of agreement; LLA, lower limit of agreement; PE, percentage error.

**Table 3:** Mean and standard deviation of Ventra’s compliance at different IAP values.

	At 5 mmHg	At 10 mmHg	At 15 mmHg	At 20 mmHg	At 25 mmHg
Low compliance (*n* = 5) (mL/mmHg)	154.39 ± 15.43	98.25 ± 16.46	84.33 ± 4.37	73.38 ± 4.71	74.33 ± 2.31
Medium compliance (*n* = 5) (mL/mmHg)	184.29 ± 34.03	123.61 ± 8.91	102.65 ± 7.11	92.89 ± 5.42	100.46 ± 8.74
High compliance (*n* = 5) (mL/mmHg)	272.12 ± 23.99	144.67 ± 11.92	108.32 ± 4.27	115.82 ± 10.13	139.49 ± 8.21

**Table 4:** Respiration-related parameters obtained in three simulations by Ventra.

	IAP_ee_ (mmHg)	IAP_ei_ (mmHg)	IAP_mean_ (mmHg)	ΔIAP (mmHg)	APV (%)	RR (rpm)	t_ins_ (s)	t_exp_ (s)	I/E (-)
1st simulation	10.71 ± 0.08	12.15 ± 0.08	11.38 ± 0.08	1.27 ± 0.11	11.16 ± 0.97	13.48 ± 2.24	2.32 ± 0.42	2.13 ± 0.61	1.09 ± 0.37
2nd simulation	10.61 ± 0.07	13.21 ± 0.16	11.91 ± 0.12	2.78 ± 0.17	23.34 ± 1.45	12.17 ± 1.32	2.52 ± 0.12	2.41 ± 0.52	1.05 ± 0.23
3rd simulation	10.13 ± 0.05	14.02 ± 0.13	12.07 ± 0.09	3.91 ± 0.14	32.39 ± 1.18	11.56 ± 0.75	2.61 ± 0.20	2.58 ± 0.27	1.01 ± 0.13

IAP_ei_, end-inspiration IAP; IAP_ee_, end-expiration IAP; APV, abdominal pressure variation; RR, respiration rate; t_ins_, inspiration time; t_exp_, expiration time.

## Data Availability

All data are available in a publicly accessible repository.

## Supplementary Materials

The following supporting information can be downloaded at: https://www.mdpi.com/article/10.3390/s24165431/s1. Figure S1. Electronic circuit of the phantom. Figure S2. Abdominal compliance tab of the GUI. Figure S3. Urine control tab of the GUI. Figure S4. Respiration tab of the GUI. Figure S5. Recordings tab of the GUI.
