# Supplementary figures and images for: An Introduction to Ventra: A Programmable Abdominal Phantom for Training, Educational, Research, and Development Purposes
Source: Sensors (Basel). 2024 Aug 22;24(16):5431. doi: 10.3390/s24165431 (PMC11359502; doi:10.3390/s24165431)

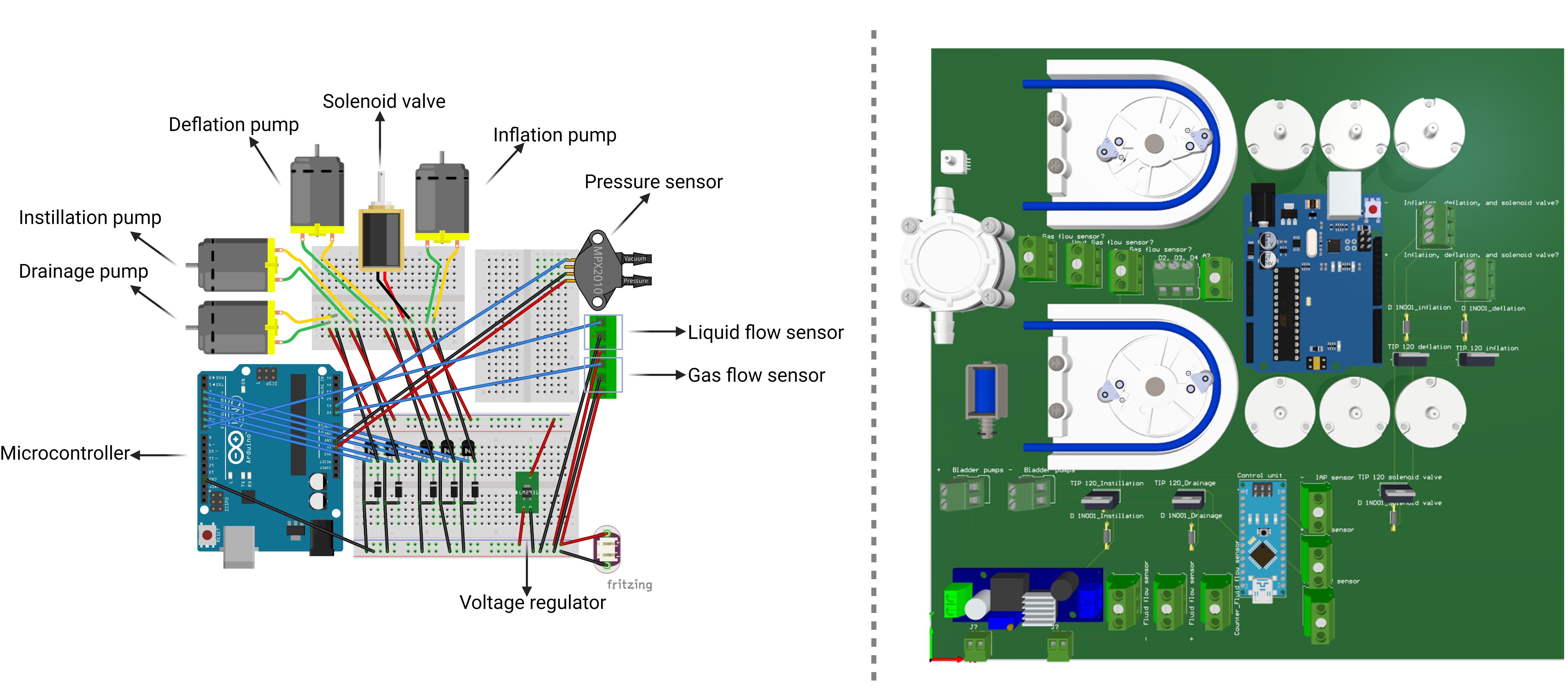

Supplement: Supplementary file 1 [file sensors-24-05431-s001.zip › Fig. S1.png]

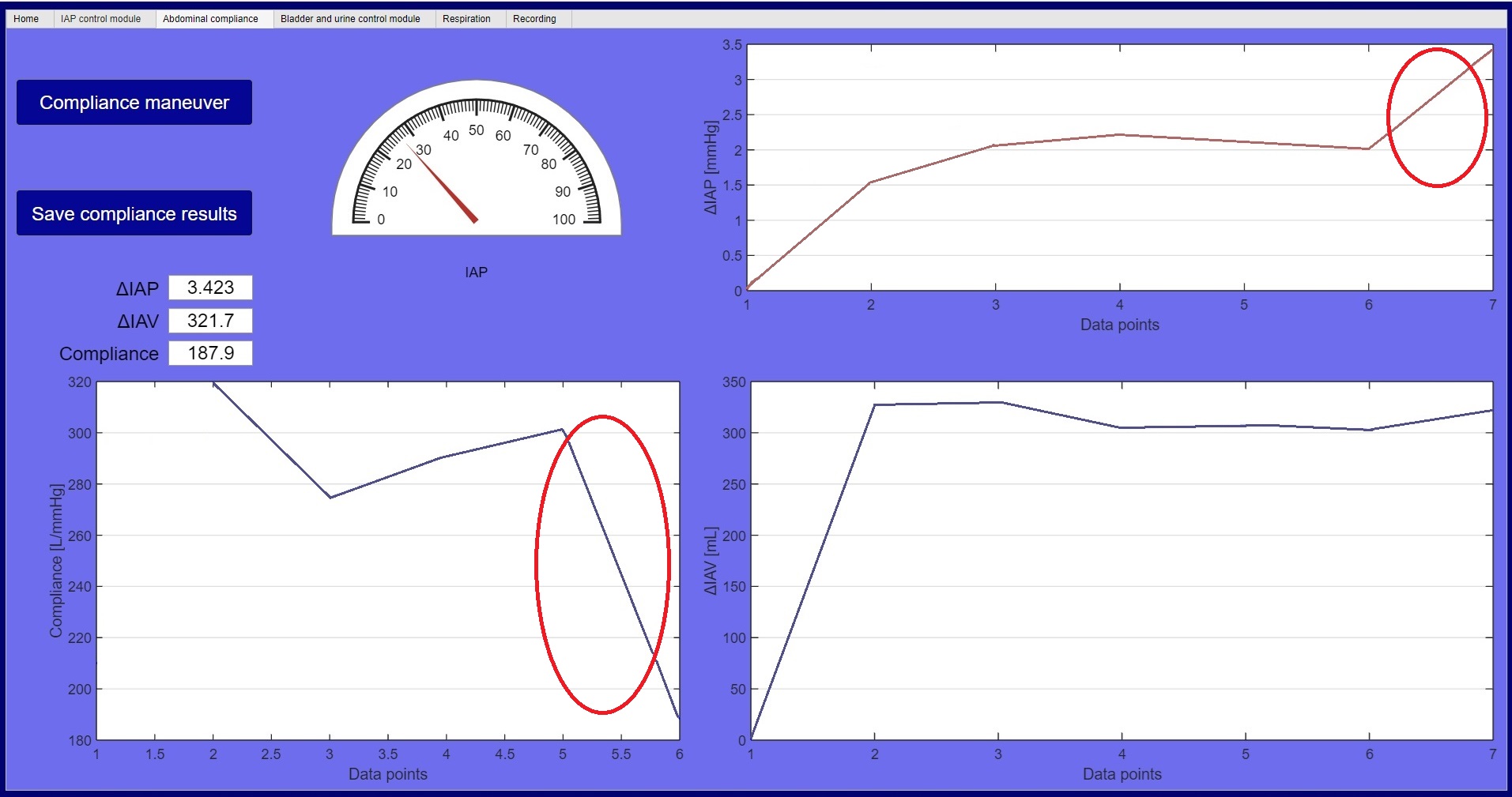

Supplement: Supplementary file 1 [file sensors-24-05431-s001.zip › Fig. S2.jpg]

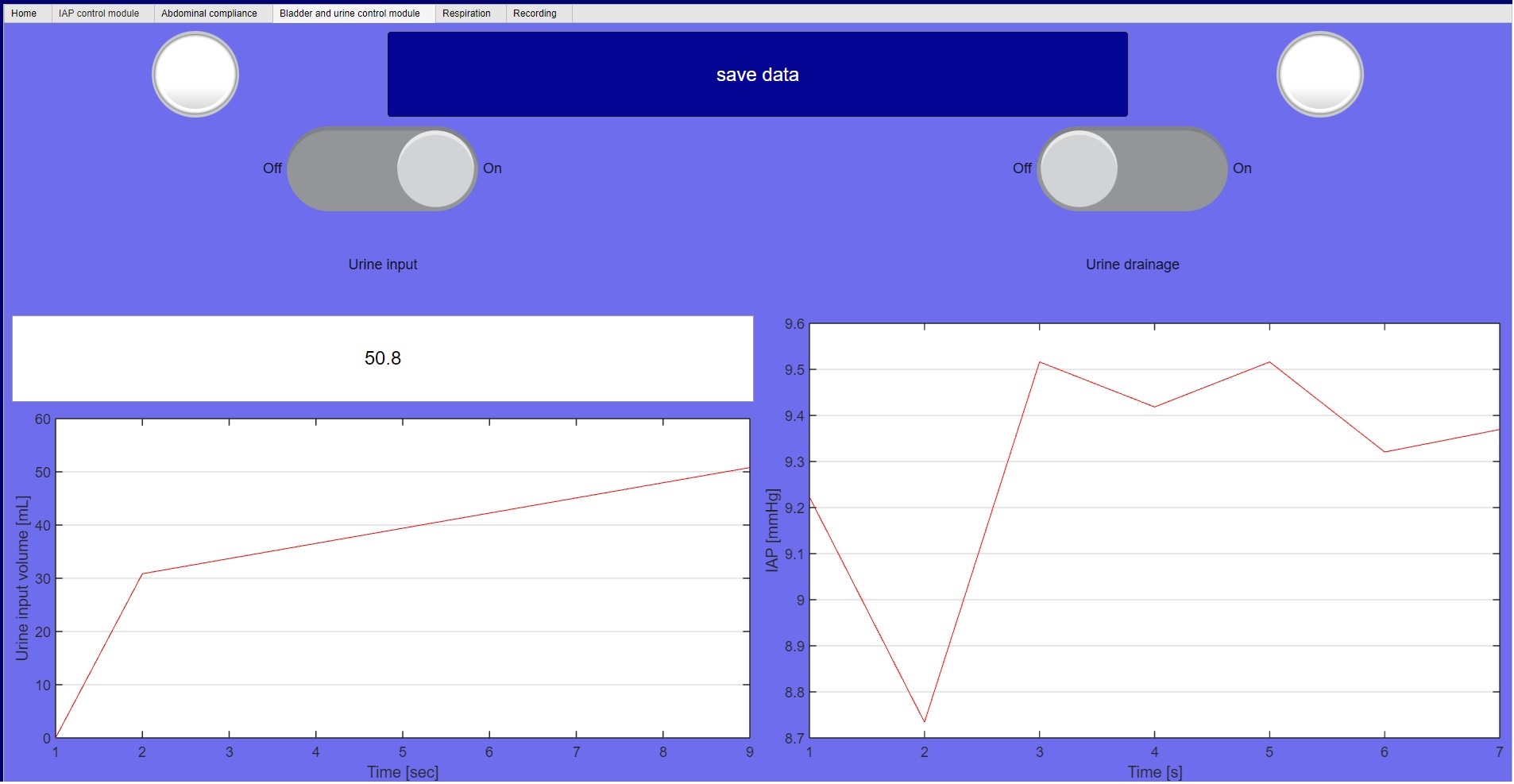

Supplement: Supplementary file 1 [file sensors-24-05431-s001.zip › Fig. S3.jpg]

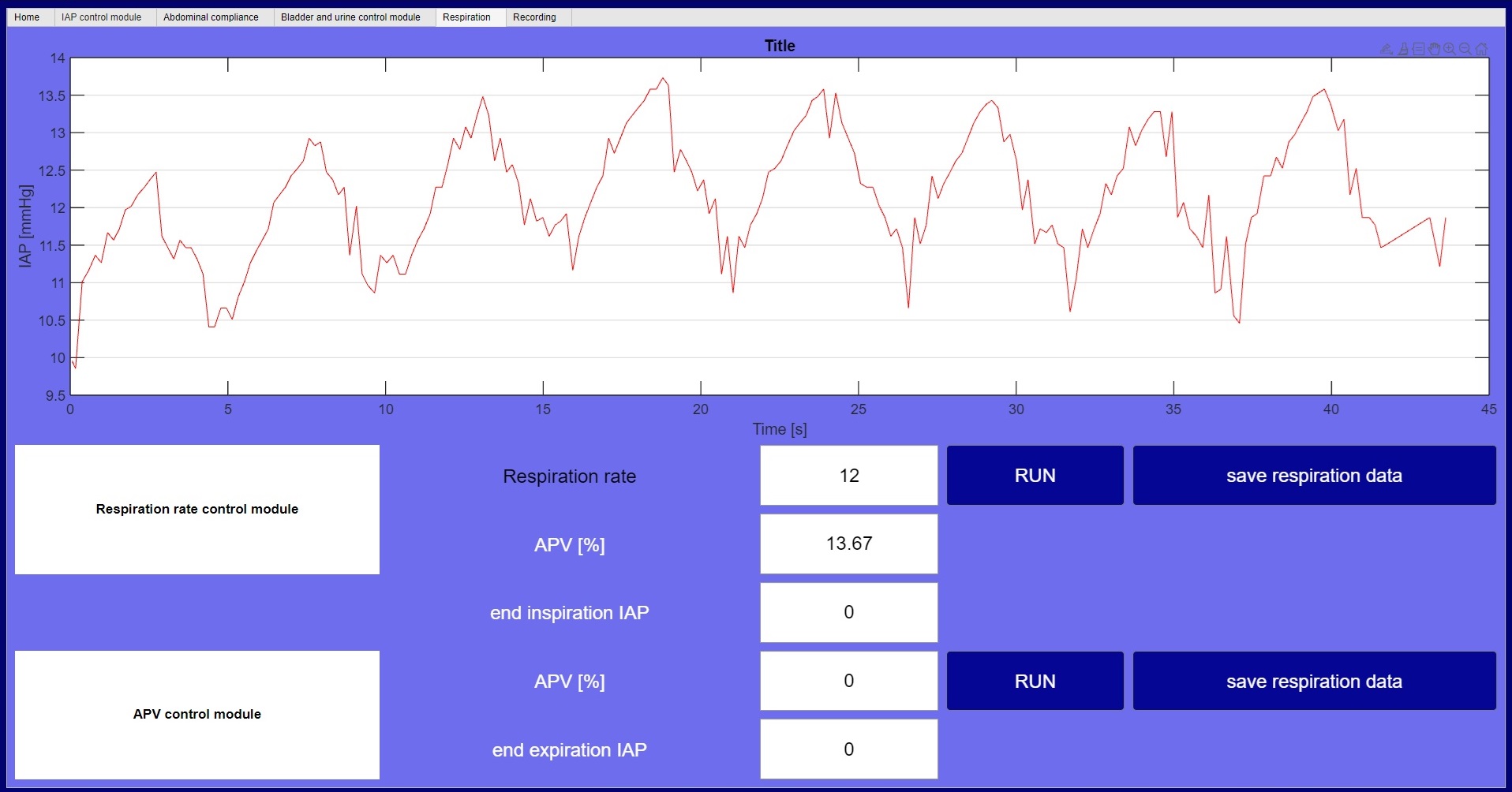

Supplement: Supplementary file 1 [file sensors-24-05431-s001.zip › Fig. S4.jpg]

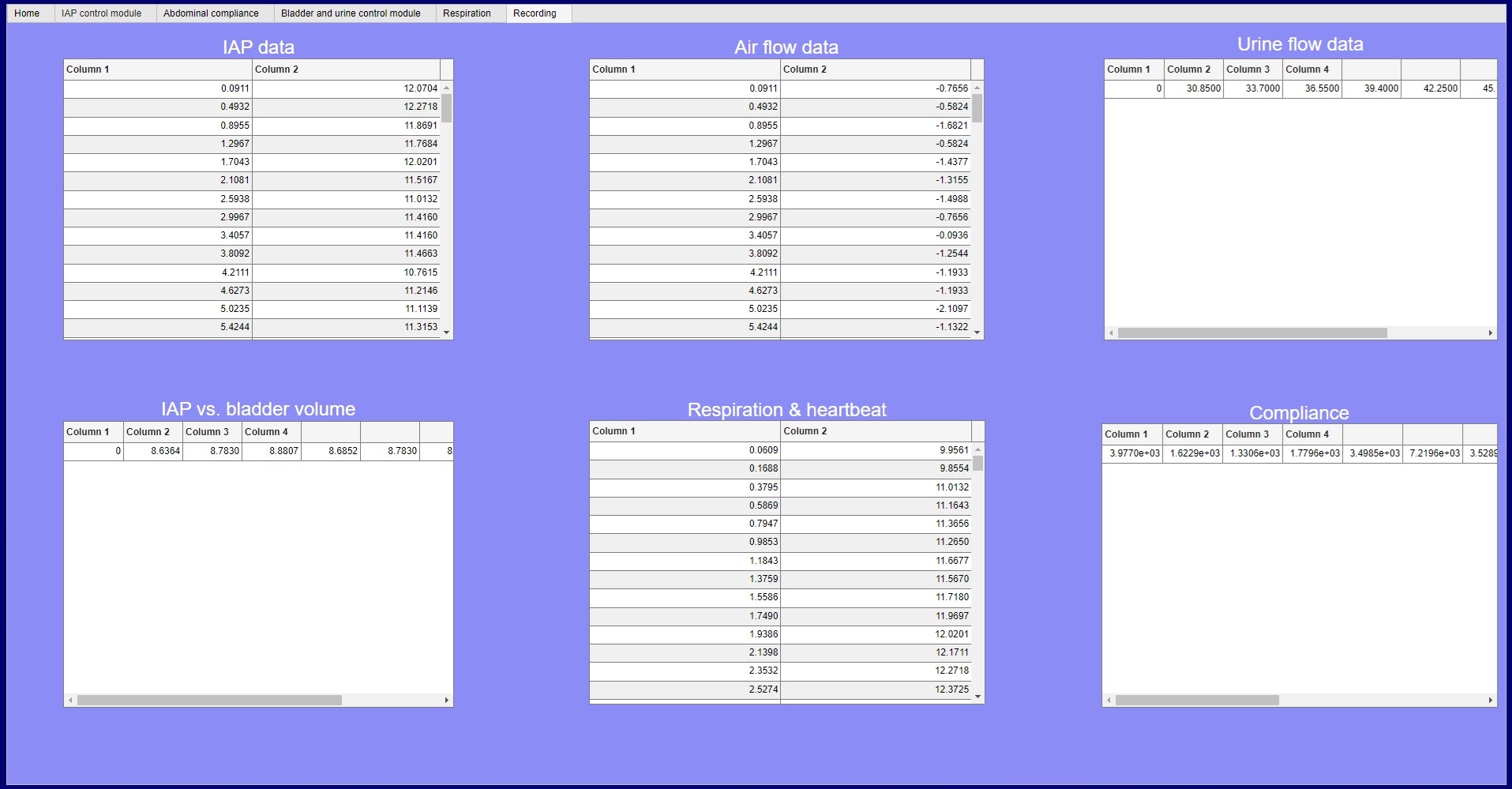

Supplement: Supplementary file 1 [file sensors-24-05431-s001.zip › Fig. S5.jpg]
